# Tumor Take Rate Optimization for Colorectal Carcinoma Patient-Derived Xenograft Models

**DOI:** 10.1155/2016/1715053

**Published:** 2016-11-23

**Authors:** Michael Gock, Florian Kühn, Christina Susanne Mullins, Mathias Krohn, Friedrich Prall, Ernst Klar, Michael Linnebacher

**Affiliations:** ^1^Department of General, Vascular, Thoracic and Transplantation Surgery, University of Rostock, Schillingallee 35, 18055 Rostock, Germany; ^2^Institute of Pathology, University of Rostock, Strempelstraße 14, 18055 Rostock, Germany

## Abstract

*Background*. For development of individualized treatment on a routine basis, transfer of patients' tumor tissue in a xenograft model (i.e., generation of patient-derived xenografts (PDX)) is desirable for molecular, biochemical, or functional analyses. Drawbacks are dissatisfactory tumor take rates, the necessity of fast tumor tissue processing, and extensive logistics demanding teamwork of surgeons, pathologists, and laboratory researchers.* Methods*. The take rates of ten colorectal cancer (CRC) tissue samples in immunodeficient mice were compared after direct cryopreservation and after a 24 h cooling period at 4°C prior to cryopreservation. Additionally, the effect of simultaneous Matrigel application on the take rates was investigated. Beside take rates, tumor growth characteristics and cell culture success were analyzed.* Results*. Tumor takes of CRC tissue samples were significantly improved after Matrigel application (8 versus 15 takes, *p* = 0.04). As expected, they diminished furthermore after 24 h cooling. Application of Matrigel could counteract this decrease significantly (2 versus 7 takes, *p* = 0.03). Cumulative take rate after cryopreservation was satisfactory (70%).* Conclusion*. Matrigel application after 24 h delay in tissue processing facilitates CRC PDX model development. These data help developing strategies for individualized tumor therapies in the context of multicenter clinical studies and for basic research on primary patient tumors.

## 1. Introduction

The milestone paper of Vogelstein et al. established a new era for understanding genetic alterations during colorectal cancer (CRC) development leading to a complete new comprehension of colorectal tumorigenesis [1]. Subsequently, major developments were accomplished in defining the main molecular classes of CRC as chromosomal and microsatellite instability and the CpG island methylator phenotype, which are recognized as key pathogenetic mechanisms [2, 3]. The analysis of these fundamental sequences led to five molecular subtypes of CRC [3, 4]. As a result of these discoveries, additional mutational analyses have revealed that in an individual cancer only a limited number of pathways are dysregulated by some “driver” mutations from a circumscribed number of about 80 candidate cancer genes [5, 6]. Transferring these findings into day-to-day clinical practice triggered the conception of targeted treatments including EGF-receptor blockade (with the prerequisite of K-Ras mutational analysis) [7, 8]. It is extremely plausible that in the near future additional “individual” molecular testing of patients' tumor tissue might become a regular step towards individual guiding and improving anticancer therapy regimens.

Tumor pieces xenografted into and expanded in immunodeficient mice, so-called patient-derived xenografts (PDX), as models of the original tumor enable, in contrast to conventional paraffin-embedded specimens, beside extensive molecular analysis also functional testing. They might thus be a central step towards truly patient orientated and individualized therapy [9–11]. Even models of metastatic diseases may be achieved as valuable research instruments for additional multidisciplinary research [12].

Yet, there are still several drawbacks hampering the use of PDX models, although standardization of processes facilitates their generation. Profound expertise and a very close teamwork encompassing several fields (surgery, pathology, and molecular and cell biology as well as animal care) are obligatory to manage logistical and technical difficulties [13]. Thus, cryopreservation of tumor samples is an effective way to resolve this situation since date and location of tumor harvesting and pathological analysis can be easily parted from tumor engrafting and subsequent molecular and functional investigations [13]. Nevertheless, facilities and corresponding expertise of cryopreservation are very limited, for the most part in nonresearch institutions. Procedures making the workflow easier, simplifying the creation, and increasing the success rate in generation of PDX models are extremely sought for.

Matrigel, a commercially available mixture of components usually found in the extracellular matrix, is well known to enhance the engrafting outcome of tumor specimen [14, 15].

We here report on a relatively easy procedure to optimize tumor take rate after xenografting of CRC specimen using Matrigel. Specifically, xenografting success rates of cryopreserved tumors with or without application of Matrigel were analyzed side by side in a consecutive series of CRCs collected* ad hoc*. In addition, we examined the feasibility of a storing routine comprising preservation of tumor specimen for one day on ice prior to cryopreservation, in order to simulate a transport of tumor samples to a facility capable of cryopreservation and/or xenografting.

## 2. Methods

### 2.1. Harvesting of Tumor Specimens and Cryopreservation

Tumor specimens (*n* = 10, primary CRC without prior chemotherapy) were received fresh from surgery. Tumor tissue cubes (3 × 3 × 3 mm) were cut from deep invasive parts with a sterile scalpel blade. For direct cryopreservation, four tumor pieces were transferred into sterile cryotubes (Greiner-Bio-One, Frickenhausen, Germany) containing 1.5 ml freezing medium (fetal calf serum containing 10% DMSO), sealed in a freezing container (Nalgene, Rochester, NY, USA), and placed immediately at −80°C. Until transplantation, tubes were kept at −80°C (for a maximum of 6 weeks), or after overnight cooling, and transferred into liquid nitrogen for longer storage periods.

For delayed cryopreservation as simulation of transport, tumor specimens were preserved on ice in a 15 ml tube in 5 ml NaCl (0.9%) for a period of 24 hours (24 h cooling) and were thereafter processed according to the above-described protocol.

For xenografting, cryopreserved tumor pieces were thawed at 37°C immediately before the xenografting procedure.

Prior written informed consent was obtained from all patients, and all procedures were approved by the Ethics Committee of the University of Rostock (reference number II HV 43/2004) in accordance with generally accepted guidelines for the use of human material.

### 2.2. Tumor Xenografting

**Figure 1:**
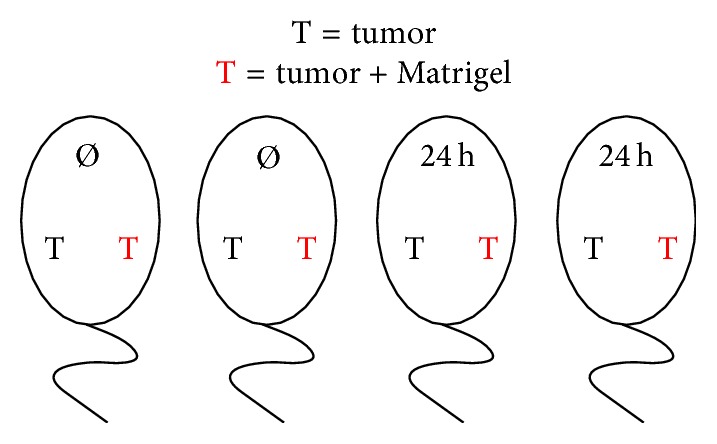
Mode of implantation of the tumor specimens in one and the same animal. Ø: no cooling; 24 h: 24 h cooling; T: tumor piece implanted (black: without and red: with Matrigel).

Experiments were performed as described in detail elsewhere [13] on female 6–8-weak-old NMRI (nu/nu) nude mice (*n* = 40) weighting 18–20 g. Tumor pieces were implanted subcutaneously bilaterally into the animals' flanks under anesthesia (ketamine-xylazine (1 : 1) mixture (1.3 *µ*l per g body weight)). Mode of implantation is depicted in Figure 1 and allows direct comparison of Matrigel application in one and the same animal at a given time point. Tumor specimens for the Matrigel group were soaked in 100 *μ*l of Matrigel (BD Biosciences, Heidelberg, Germany) for 20 seconds at 20°C immediately prior to xenografting.

Mice were kept in the animal facilities of the medical faculty of the University of Rostock and maintained in specified pathogen-free conditions. Animals were exposed to 12 h light/12 h darkness cycles and standard food and water including antibiotics (Cotrimoxazole)* ad libitum*. Their care and housing were in accordance with guidelines as put forth by the German Ethical Committee and the Guide for the Care and Use of Laboratory Animals (Institute of Laboratory Animal Resources, National Research Council; NIH Guide, volume 25, number 28, 1996). The protocol was approved by the Committee on the Ethics of Animal Experiments of the University of Rostock (Landesamt für Landwirtschaft, Lebensmittelsicherheit und Fischerei Mecklenburg- Vorpommern; Thierfelder Str. 18, 18059 Rostock, Germany; permit number: LALLF M-V/TSD/7221.3-1.1-071-10). Growth of tumors to volumes of 1–1.5 cm^3^ was taken as evidence of successful xenografting, and the animals were then sacrificed for collection of tumor tissues for further studies.

### 2.3. Verification of the Human Origin of the Harvested Tumors and Genetic Fingerprint

For verification of the human origin of our harvested tumors, a human specific PCR was performed by amplification of a portion of the human mitochondrial cytochrome b gene as previously described [16]. Briefly, the reaction mixture (25 *μ*l) contained 25 ng of gDNA, 0.1 mM of each primer (L15674: TAGCAATAATCCCCATCCTCCATATAT, H15782: ACTTGTCCAATGATGGTAAAAGG), 200 *μ*M dNTPs, 1x standard reaction buffer, and 0.1 U Taq DNA polymerase (Bioron, Ludwigshafen, Germany). PCR was performed in a standard thermal cycler for 40 cycles of 30 s at 96°C, 40 s at 59°C, and 1 min at 72°C. Products were separated on a 1% agarose gel and results were scored positive with the appearance of a band of 157 bp.

The identity of each cell line was checked by a short tandem repeat analysis at 9 different loci (D5S818, D13S317, D7S820, D16S539, vWA, TH01, TPOX, CSF1PO, and amelogenin for sex determination). Multiplex PCR amplicons were separated by capillary electrophoresis and analyzed using GeneMapperID software from Life Technologies (Darmstadt, Germany).

### 2.4. Cell Line Generation from PDX Material

Cell line generation has previously been described in detail [17]. Briefly, PDX tumors were minced; cells were released from surrounding tissue by scraping and passage through a nylon mesh (100 *µ*m) to obtain single cell suspensions. This was seeded on collagen-coated 6-well plates in medium supplemented with 10% FCS, 200 mM L-glutamine, antibiotics (penicillin G 10.000 IU/L; streptomycin 130 mg/L), and antimycotics (Amphotericin B 6 mg/L). Plates were incubated at 37°C in a humidified atmosphere of 5% CO_2_. All cell culture reagents were obtained from Pan Biotech (Aidenbach, Germany); antibiotics and antifungal agents were from the pharmacy of the university hospital Rostock. Medium was regularly changed and cells were passaged into 25 cm^2^ culture flasks when tumor cell growth was observed.

### 2.5. Histopathological Analysis

Histopathological examinations of the primary tumors were done according to standard protocols for surgical pathology reports of CRCs [18]. Primary and PDX tumor tissue were embedded in paraffin and 4 *μ*m H&E sections were made. Morphology of tumor architecture, growth pattern, and cytological features of primary and xenograft tumors were examined and compared.

### 2.6. Statistical Analysis

A Chi-square test (one or two sided) was used to test whether differences between two groups were significant. Analyses were performed using GraphPad Prism 5 software and *p* values <0.05 were considered significant.

## 3. Results

In this study, 10 colorectal adenocarcinomas were consecutively collected. Localization and TNM staging as well as patients data are presented in Table 1. In addition, length of cryopreservation (40 to 293 days) prior to implantation is depicted in Table 2.

Vital tumor pieces from the invasive front were first cryoconserved and subsequently engrafted into nude mice. A side-by-side comparison was performed with tumor pieces immediately frozen after the tissue preparation and a second set of tumor pieces left at approximately 4°C for a 24 h cooling period before cryopreservation. A second systematic comparison was performed by either briefly soaking the tumor pieces in Matrigel or not before subcutaneously implanting soaked and unsoaked pieces of the same tumor into different sides of the same animals. The overall outcome is given in Table 2.

### 3.1. Overall Xenografting Success Rate

The general analysis of the 10 CRC included into this study revealed that outgrowing PDX were obtained in seven out of the ten cases, thus summing up to a 70% take rate (actual results are summarized in Table 2).

### 3.2. Effect of Delayed Cryopreservation

For simulation of a time delay in the tissue handling process such as transport and the like, tumor tissue was preserved for a 24 h period on ice (24 h cooling). Thereafter, cryopreservation was done according to the protocol. Take rate analysis of this group (24 h cooling) compared to the group of direct cryopreservation (no cooling) revealed a profoundly diminished take rate which barely failed to reach statistical significance (without Matrigel: *n* = 6 takes with no cooling versus *n* = 2 takes with 24 h cooling; *p* = 0.06).

### 3.3. Effect of Matrigel Application

In order to allow best analysis of the effects of Matrigel application, animals received tumor implantation in a bilateral manner: one side without and the other side with presoaking in Matrigel. In both groups (no cooling and 24 h cooling) combined, application of Matrigel resulted in a significantly improved tumor take rate (*n* = 15 takes with Matrigel versus *n* = 8 takes without Matrigel; *p* = 0.04 Chi-square test). Analysis of the group of directly cryopreserved tumors (no cooling) showed that application of Matrigel had only a minor, statistically not significant effect on take rate (no cooling with Matrigel *n* = 8 takes versus no cooling without Matrigel *n* = 6 takes; *p* = 0.25 Chi-square test).

More importantly, in the group of delayed cryopreserving (24 h cooling), application of Matrigel ameliorated tumor take rate significantly (24 h cooling with Matrigel *n* = 7 takes, versus 24 h cooling without Matrigel *n* = 2 takes; *p* = 0.03 Chi-square test).

### 3.4. Morphological Analysis of Histopathology, Molecular Analysis, and Demonstration of Tumor Cell Viability

**Figure 2:**
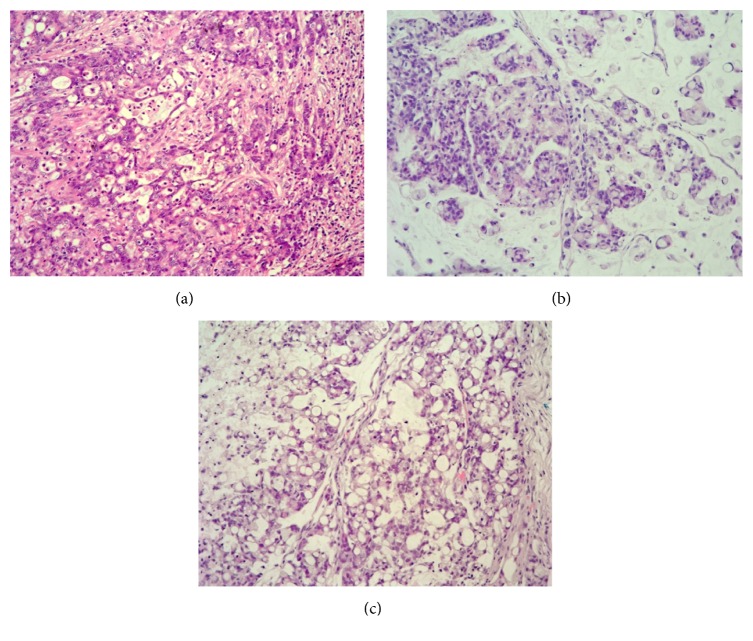
Tumor morphology (20x). Exemplary HE stains from HROC131. (a) Primary tumor; (b) the PDX generated according to the no cooling and (c) the PDX generated according to the 24 h cooling protocol. (b) and (c) are from the cases with the addition of Matrigel. Tumor architecture, growth pattern, and cytological features of the primary tumor are well preserved in the PDX.

Analysis performed by an expert pathologist (FP) showed that, histologically, the PDX closely resembled their primaries (Figure 2). Additionally, the group of delayed cryopreserving showed similar tumor architecture, growth pattern, and cytological features compared to the group of directly cryopreserved tumors (Figure 2(b) versus Figure 2(c)).

In addition, PCR studies amplifying part of the human mitochondrial cytochrome b gene confirmed the human origin of the PDX tumors (data not shown).

**Figure 3:**
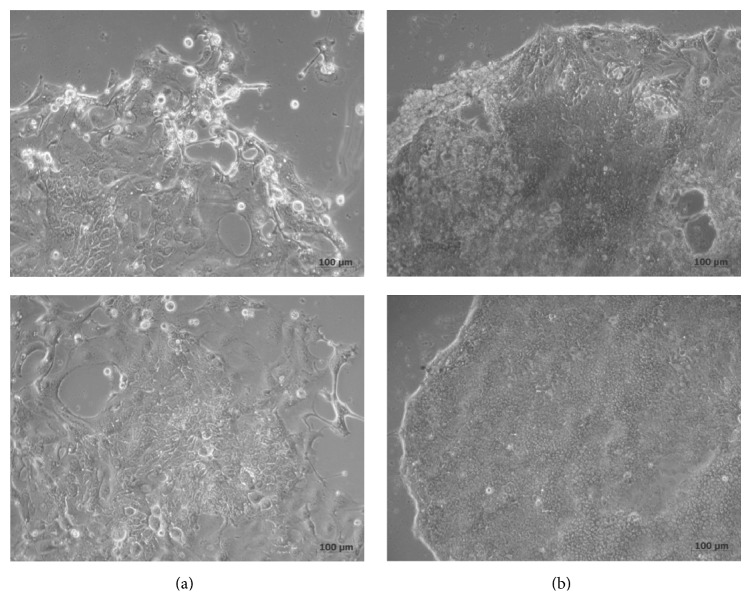
Depicted are the two successfully established cell lines form this series in passage number 1. Two different areas are displayed to demonstrate the minor morphological differences. (a) HROC107. Note the hem of obviously dying cells in the upper left picture. In the colony center (also of the lower left picture), a population of cells with a copper-stone phenotype can be found which dominated the culture in subsequent passages. (b) HROC131. Similar to HROC107, areas with a larger cell type can be observed in the upper right picture. Smaller cells (displayed in the lower right picture), again with a copper-stone phenotype, took over in the culture in later passages, too.

Subsequent to the PDX generation, we also routinely generated primary cell cultures out of the established PDX. This procedure was successful in all cases with no observed differences between the no cooling and 24 h cooling PDX. Thus, the viability of the PDX-derived tumor cells was obviously not affected from the 24 h cooling procedure nor from the presence or absence of Matrigel at the time of tumor tissue implantation. Of note, in two cases, permanent cell lines were generated from the PDX primary cultures of the 24 h delay group (i.e., HROC107 and HROC131) (Figure 3). Fingerprint analyses of these two cell lines again confirmed human origin as well as genetic identity with the patients' tumors and PDX models they were generated from (Table 3(a)). A basic analysis of relevant mutations for CRC and further genetic features performed side by side with the PDX models and the primary tumors also revealed no differences (Table 3(b)). Accordingly, HROC107 could be classified as a sporadic standard type CRC whereas HROC131 is sporadic microsatellite instable (Table 3(b); classification according to [3]) CRC.

## 4. Discussion

Generating models of individual human tumors is an important step towards truly individualized therapy since it facilitates functional testing in addition to pure molecular analysis [9–11]. However, creation of these models is still challenging. On the one hand, the success rate for generation of individual permanent cell lines for CRC is reported to rarely exceed 10% [19], and on the other hand, exquisite expertise and a close teamwork of surgery and pathology as well as molecular and cell biology and animal care are involved in PDX establishment [13, 20, 21]. Recently, we could show that xenografting of prior cryopreserved tumor specimen may be equally successful as xenografting of fresh tumor material [13]. This is an approach to minimize logistical and timing problems when fresh tumor tissue is being engrafted. The pure tumor harvesting can thus be spatiotemporally separated from the xenografting [13] disentangling the logistical problems. Nevertheless, a close teamwork of surgery, pathology, and laboratory is mandatory for fast processing of tumor samples. Capability of cryopreservation of tumor samples is limited to a low number of institutions and therefore new and optimized methods are required to overcome these restrictions.

In order to improve the “take rate” of xenografted tumor specimen, we here systematically analyzed effects of soaking the tumor specimens in Matrigel prior to implantation. This approach was previously reported in PDX generation from pancreatic and other carcinomas [21, 22], but a systematical and comparative approach has so far not been reported. Our mode of implantation allows such a systematical and direct comparison of engraftment with and without Matrigel in one and the same animal at a given time point thus allowing a concrete analysis and minimizing potential bias from the experimental animal side.

With the intention to simulate a logistical delay between tumor surgery and the cryopreservation procedure, as would necessarily be the case when tumor tissue is acquired from distant institutions, especially nonuniversity outpatient clinics, we analyzed the effects of Matrigel in an additional group with a 24-hour 4°C cooling delay before cryopreservation. Analysis of delayed xenografting after this 24 h cooling period of specimen storage on ice showed a considerable reduction of tumor take rate. Though not significant, however showing a strong trend (*p* = 0.06), this result could be explained by biological degradation processes impairing cell viability and fitness.

On the other hand, we could show that application of Matrigel resulted in a significantly improved tumor take rate in this group of delayed xenografting. Contrary to this, Matrigel pretreatment of directly cryopreserved tumors had only a minor effect on take rate.

Therefore, addition of Matrigel seems to counteract the deterioration of delayed cryopreserving after a 24 h cooling period on ice. Interestingly, our cumulative take rate was 70% and was as satisfying as in our previously reported series with a cumulative take rate of 71% [13]. In accordance with these previous findings, the length of the cryopreservation period had no influence on take rate (*p* = 0.9489).

This addendum to our cryopreservation protocol may help to overcome the well-known logistical limits of individual tumor model generation especially if site of tumor harvesting and place of cryopreservation (e.g., hospital and laboratory) are geographically distant and thus may allow preclinical studies or individual testing of targeting therapies in patient tumor tissue of centers without the ability of the complex cryopreservation procedure on site.

Since primary cell cultures could also successfully be established from the PDX tissues obtained, one can safely assume that cell viability is not generally or at least not sustainably impaired from the delay in cryopreservation. Sustained cellular fitness is further corroborated by the fact that two permanent cell lines (HROC107 and HROC131) were successfully established from these primary cultures.

At last, the option of a delayed cryopreservation will greatly facilitate selection and establishment of different tumor models, especially if low passage PDX (or cell line) models are mandatory. This might be an important step towards more individual guided and improved anticancer therapy regimens

## Figures and Tables

**Table 1:** 

Tumor ID	Age	Sex	TNM	Localization
HROC107	74 y	Male	pT3pN2cM1 G2 R0 L1 V0	Sigmoid
HROC118	70 y	Male	pT4pN1cM0 G2 R0 L0 V1	Ascending colon
HROC119	72 y	Male	pT3pN0cM0 G3 R0 L1 V0	Coecum
HROC122	80 y	Male	pT4pN0cM0 G3 R2 L0 V1	Sigmoid
HROC123	74 y	Male	T4 N2 M0 G3 R0 L0 V0	Descending colon
HROC125	84 y	Female	pT3pN1cM0 G2 R0 L0 V0	Sigmoid
HROC129	76 y	Female	pT3pN1cM0 G2R0L1V0	Transversal colon
HROC130	60 y	Male	pT3pN1cM1 G3R2L0V0	Sigmoid
HROC131	73 y	Female	pT3pN1cM0 G3R0L0V0	Ascending colon
HROC135	75 y	Male	pT3pN1cM0 G3R0L0V0	Ascending colon

**Table 2:** 

Tumor ID	Outcome		Days frozen
	No cooling	24 h cooling	
HROC107	M1(+/+); M2(+/−)	M3(−/+); M4(−/+)	293
HROC118	M1(−/+); M2(−/−)	M3(−/+); M4(−/+)	174
HROC119	M1(−/−); M2(−/−)	M3(−/−); M4(−/−)	154
HROC122	M1(−/−); M2(−/−)	M3(−/−); M4(−/−)	130
HROC123	M1(+/−); M2(−/−)	M3(−/−); M4(−/−)	130
HROC125	M1(−/−); M2(−/−)	M3(−/−); M4(−/−)	74
HROC129	M1(+/+); M2(−/+)	M3(−/−); M4(−/−)	61
HROC130	M1(−/+); M2(−/−)	M3(−/−); M4(−/−)	57
HROC131	M1(+/+); M2(+/+)	M3(+/+); M4(+/+)	54
HROC135	M1(−/+); M2(−/−)	M3(−/−); M4(−/+)	40
Ø Matrigel	6/20	2/20	
With Matrigel	8/20	7/20	

A total of 4 mice were implanted on both flanks with human CRC tissue samples. M denotes individual animals xenografted; the outcome is given in parentheses as index + (outgrowing tumor) or index − (no outgrowth). Tumor tissues presoaked with Matrigel are indicated in underlined signs. The overall number of successful sites with and without the addition of Matrigel is given for the total of 20 sides implanted.

**(a):** 

ID	vWA	TH01	TPOX	CSF1 PO	D5S818	D13S317	D7S820	D16S539	Sex
HROC107	16, 17	8, 9	10, 11	10, 12	11, 13	8	10, 11	10, 13	m
HROC131	16	10	8	11	12, 13	12	11, 13	8, 12	f

**(b):** 

ID	Molecular type	Ploidy status	Mutations		K-Ras	N-Ras	H-Ras	PIK3CA	B-Raf	CIMP-number	MSI - status
			p53	APC							
HROC107	spStd	Aneuploid	Ex8	mut	G12D	wt	wt	E542K	wt	2	MSS
HROC131	spMSI-H	Aneuploid	n.a.	n.a.	wt	wt	wt	wt	mut	5	MSI-H

(a) Fingerprint analysis of cases HROC107 and HROC131. The alleles of nine classical markers are displayed. No differences were observed between the original tumor and both the PDX models and the tumor cell lines generated from the 24 h PDX models.

(b) The results of the molecular analysis of cases HROC107 and HROC131 (according to [3]) are displayed. Mutations in the CRC-relevant target genes p53, APC, K-, N-, and H-Ras, PIK3CA, and B-Raf were analyzed. Together with the CIMP and MSI analysis results, the underlying molecular type could be identified as spSTD for HROC107 and spMSI-H for HROC131.

ID: pseudonym of case, sex: result of the amelogenin marker analysis, m: male, f: female, sp: sporadic, Std: standard type, Ex8: exon number 8, mut: mutated, wt: wild type, n.a.: not analyzed, CIMP: CpG island methylator phenotype, MSI: microsatellite instability, MSS: microsatellite stable, MSI-H: high grade microsatellite instable.
